# Tolerability and Safety Assessment of Adjuvant Chemoradiotherapy with S-1 after Limited Surgery for T1 or T2 Lower Rectal Cancer

**DOI:** 10.3390/cancers16193360

**Published:** 2024-09-30

**Authors:** Norikatsu Miyoshi, Mamoru Uemura, Shingo Noura, Masayoshi Yasui, Junichi Nishimura, Mitsuyoshi Tei, Chu Matsuda, Shunji Morita, Akira Inoue, Hiroki Tamagawa, Yukako Mokutani, Shinichi Yoshioka, Makoto Fujii, Shinya Kato, Yuki Sekido, Takayuki Ogino, Hirofumi Yamamoto, Kohei Murata, Yuichiro Doki, Hidetoshi Eguchi

**Affiliations:** 1 Department of Gastroenterological Surgery, Graduate School of Medicine, Osaka University, Osaka 565-0871, Japan; skato@gesurg.med.osaka-u.ac.jp (S.K.); ysekido@gesurg.med.osaka-u.ac.jp (Y.S.); togino@gesurg.med.osaka-u.ac.jp (T.O.); hyamamoto@gesurg.med.osaka-u.ac.jp (H.Y.); ydoki@gesurg.med.osaka-u.ac.jp (Y.D.); heguchi@gesurg.med.osaka-u.ac.jp (H.E.); 2 Department of Innovative Oncology Research and Regenerative Medicine, Osaka International Cancer Institute, Osaka 541-8567, Japan; 3 Department of Surgery, Sakai City Medical Center, Sakai 593-8304, Japan; snoura0410@gmail.com; 4 Department of Gastroenterological Surgery, Osaka International Cancer Institute, Osaka 541-8567, Japan; myasui@oici.jp (M.Y.); jnishimura@oici.jp (J.N.); 5 Department of Surgery, Osaka Rosai Hospital, Osaka 252-3561, Japan; mtei@osakah.johas.go.jp; 6 Department of Surgery, Osaka Police Hospital, Osaka 543-0035, Japan; chu0710pinefield@aol.com; 7 Department of Surgery, Itami Municipal Hospital, Itami 664-0015, Japan; shun4morita@gmail.com; 8 Department of Gastroenterological Surgery, Osaka General Medical Center, Osaka 558-8558, Japan; inoue_medical@yahoo.co.jp; 9 Department of Gastroenterological Surgery, Otemae Hospital, Osaka 540-0008, Japan; tamagawa.hiroki@mbl.co.jp; 10 Department of Surgery, Higashiosaka City Medical Center, Higashiosaka 578-8588, Japan; yukakohenry@yahoo.co.jp; 11 Department of Surgery, Yao Municipal Hospital, Yao 581-0069, Japan; room335@mars.dti.ne.jp; 12 Department of Mathematical Health Science, Graduate School of Medicine, Osaka University, Osaka 565-0871, Japan; m.fujii@sahs.med.osaka-u.ac.jp; 13 Department of Gastroenterological Surgery, Kansai Rosai Hospital, Amagasaki 660-0064, Japan; kmuratajp@yahoo.co.jp

**Keywords:** adjuvant chemoradiotherapy, early rectal cancer, local excision

## Abstract

**Simple Summary:**

The study evaluated the long-term outcomes of chemoradiotherapy (CRT) with S-1 after limited surgery for T1 or T2 lower rectal cancer. The 3-year and 5-year relapse-free survival rates were 90.17% and 85.87%, respectively, showing favorable outcomes for T1 cancer patients, with effective anal function preservation. However, further treatment is needed to improve outcomes for T2 cancer patients.

**Abstract:**

Background: The short-term outcomes of chemoradiotherapy (CRT) with S-1 (a combination of tegafur, gimeracil, and oteracil) following limited surgery for patients with T1 or T2 lower rectal cancer have shown encouraging results. Objectives: This study was designed to delve deeper into the long-term outcomes of CRT with S-1 after limited surgery, with the goal of evaluating both the long-term efficacy and potential risks associated with this treatment approach in patients diagnosed with T1 or T2 lower rectal cancer. Methods: This was conducted as a multicenter, single-arm, prospective phase II trial. The patient population consisted of individuals clinically diagnosed with either T1 or T2 lower rectal or anal canal cancer, with a maximum tumor diameter of 30 mm and classified as N0 or M0. Patients underwent local excision or endoscopic resection. After surgery, CRT with S-1 was administered to patients meeting several criteria, including the confirmation of well-differentiated or moderately differentiated adenocarcinoma, negative surgical margins, submucosal invasion depth of ≥1000 µm, and high tumor-budding grade (2/3). The primary endpoint of this study was relapse-free survival, while secondary endpoints included local recurrence-free survival, overall survival, anal sphincter preservation rate, and safety. Results: A total of 52 patients were included, with pathological diagnoses revealing T1 in 36 patients and T2 in 16 patients. The 3-year and 5-year relapse-free survival rates were 90.17% and 85.87%, respectively. The 3-year and 5-year local recurrence-free survival rates were 90.17% and 88.07%, respectively, while the 3-year and 5-year overall survival rates were 94.03% and 91.94%, respectively. Conclusions: CRT with S-1 after limited surgery for T1 lower rectal cancer demonstrated favorable outcomes in terms of recurrence, survival, and local control rates while effectively maintaining anal function in patients. However, further treatment approaches may be necessary to improve outcomes for patients diagnosed with stage T2 lower rectal cancer

## 1. Introduction

The standard treatment for rectal cancer is total mesorectal excision (TME), including procedures such as low anterior resection, intersphincteric resection, or abdominoperineal resection [1,2]. In some cases, transanal TME may also be utilized to ensure safety and maintain radicality [3,4]. However, this approach is associated with a high operative morbidity rate, significantly impacting functional outcomes and reducing postoperative quality of life due to the decline in anal function [5,6,7].

Local excision has been explored as an alternative therapy for patients with early-stage rectal cancer who are not suitable candidates for radical surgery or who have a strong desire to preserve their sphincter function. The primary issue with rectal cancer local excision is the risk of recurrence. Local excision by itself has reported recurrence rates ranging from 0 to 22% for T1 lesions [8,9,10,11,12,13] and up to 37% for T2 lesions [14,15,16], resulting in poorer long-term oncological outcomes and a higher risk of cancer-related mortality than TME [17,18]. However, advances in local excision methods, alongside preoperative or postoperative radiation or chemoradiotherapy (CRT), have improved local control rates to 0–8% in pT1 disease and 0–19% in pT2 disease while preserving patient quality of life [14,15,16,17,19,20].

The National Comprehensive Cancer Network (NCCN) [2] recommends adjuvant therapy options for patients diagnosed with pT2 (the tumor invades the muscularis propria) or pT1 (the tumor invades the submucosa) NX with high-risk features following transanal local excision, including transabdominal resection and CRT. High-risk features, in addition to positive resection margins, include lymphovascular invasion, poorly differentiated tumors, and submucosal invasion of the lower third of the submucosa after complete local excision. Similarly, the 2019 guidelines from the Japanese Society for Cancer of the Colon and Rectum (JSCCR) [21] classify submucosal invasive colorectal cancer lesions as high risk for lymph node metastasis if they exhibit any of the following characteristics: submucosal invasion depth >1000 μm, positive lymphovascular invasion, poorly differentiated adenocarcinoma, signet-ring cell carcinoma, mucinous carcinoma, and/or the presence of tumor budding at the invasive front, classified as Grade 2 or 3. These guidelines also recommend transabdominal resection, including TME, as the preferred treatment for pT1 lesions with high-risk features and pT2 lesions [21].

As indicated in the guidelines, radical surgery remains the standard treatment for pT1 lesions with high-risk features and pT2 lesions, although the results of ongoing clinical trials are eagerly awaited. Local excision combined with CRT is a meaningful approach if both oncological clearance and quality of life can be achieved. We previously reported on the short-term outcomes of CRT with S-1 after limited surgery for T1 or T2 lower rectal cancer, demonstrating its promising safety and feasibility [22]. In the present study, we aimed to report the final results and provide a comprehensive evaluation of the long-term efficacy and potential risks of this combined treatment method in patients with T1 or T2 lower rectal cancer.

## 2. Patients and Methods

### 2.1. Methods

The Clinical Study Group of Osaka University, Colorectal Group, designed this prospective, multicenter, single-arm phase II trial (UMIN 000007184), which was conducted in compliance with the Declaration of Helsinki and its amendments to ensure the highest level of patient protection. The protocol received approval from the institutional review boards of all participating hospitals (No. 12018-2, 18545-2), and written informed consent was obtained from each patient prior to enrollment. Patient enrollment took place at nine hospitals from 7 June 2012 to 26 February 2017.

### 2.2. Study Cohort and Eligibility

Patients with clinically diagnosed T1 or T2 lower rectal (the proximal boundary of the lower rectum was defined as the second Houston valve) or anal canal cancer, with a maximum tumor diameter of 30 mm and classified as cN0 and cM0, were included. Anal cancer was excluded in the current analysis. The diagnosis involved CT scans of the thorax and abdomen to assess the regional lymph nodes and distant metastases. Patients who were eligible underwent local transanal excision, endoscopic mucosal resection, or endoscopic submucosal dissection. The inclusion criteria required patients to be over 20 years old, have a performance status (ECOG-PS) of 0–1, and have no severe comorbidities. Exclusion criteria consisted of previous pelvic irradiation, concurrent malignancies within the past five years, and unsuitability for subsequent CRT or surgery.

### 2.3. Surgical and Postoperative Treatment Protocol

Surgeries were performed under general, spinal, or local anesthesia at the discretion of the surgeon. The surgical approach was flexible, allowing for both endoscopic and non-endoscopic methods. Surgeons decided whether to suture the rectal wall defect or leave it open. Postoperative CRT was planned to begin within 12 weeks of surgery, with radiotherapy administered using megavoltage equipment (>6 MV) and computed tomography-based planning. S-1 was administered orally twice daily on specific days over a 33-day period, with dosages adjusted based on the body surface area. Radiotherapy was delivered five days a week, excluding weekends, with a total dose of 45 Gy administered in 25 fractions. The clinical target volume included the primary tumor bed, mesorectum, and specific lymph nodes with anatomically defined borders [23]. The planning target volume accounted for organ motion and setup errors, and a lower prophylactic radiation dose of 45 Gy was used for the mesorectum without a tumor bed boost.

### 2.4. Follow-Up and Evaluation

The patients underwent regular follow-ups, including physical examinations, assays for carcinoembryonic antigen and carbohydrate antigen 19–9 measured via blood tests, digital rectal examinations, CT scans, and colonoscopies performed according to the guidelines laid out by the JSCCR [24], over a five-year period post-CRT. Suspected recurrences were further evaluated, with radical surgery performed for resectable recurrences. The main objective of this research was to thoroughly assess the five-year relapse-free survival (RFS) rate, which serves as a key indicator for long-term outcomes in patients. This measure allows for a clear understanding of how effectively the treatment prevents recurrence over a significant period. In addition to this primary endpoint, several secondary objectives were also examined to provide a more comprehensive view of the treatment’s impact. These secondary endpoints included the rate of treatment completion, which reflects patient adherence and tolerance to the prescribed regimen, and the relative dose intensity, which offers insights into whether patients were able to receive the intended dosage without significant reductions. Safety was another critical secondary endpoint, and it was evaluated by monitoring adverse events and any complications that arose during or after surgery, ensuring that both short- and long-term risks were properly addressed. Other secondary endpoints included the anal sphincter preservation rate, which is particularly relevant in maintaining quality of life post-surgery, as well as the preservation of anal function itself. Furthermore, the five-year local recurrence-free survival (LRFS) rate and the five-year overall survival (OS) rate were tracked to provide additional metrics for evaluating patient outcomes. In the case of RFS, the period used for the analysis was calculated from the date of surgery to the date of recurrence or death from any cause, whichever event occurred first, ensuring that the timing of these outcomes was precisely measured. For patients who did not experience recurrence or were not diagnosed with cancer other than recurrence, the period was censored on the date of the last confirmed survival without recurrence (last recurrence-free survival confirmation date: for inpatients, the investigation date; for outpatients, the latest outpatient visit date or the most recent examination date). OS was measured from the date of surgery to the date of death from any cause, with the period being censored on the last confirmed survival date for patients who were still alive. In the case of patients lost to follow-up, the censoring period was based on the last confirmed date of survival prior to the loss of contact. LRFS rates were evaluated according to a predetermined schedule, with follow-up examinations and observations carried out every three months during the first three years after surgery and subsequently every six months to monitor the presence or absence of local recurrence and to calculate the cumulative LRFS rates. The date of metastasis or local recurrence was defined as the earliest confirmed date when these events could be verified by imaging studies. The rate of anal sphincter preservation was documented, taking into account the proportion of cases where the anal sphincter was preserved during local excision, including both surgical and endoscopic methods. Adverse events were recorded and analyzed based on the National Cancer Institute’s Common Toxicity Criteria (version 4.0), and anal function was assessed at six-month intervals, using both the Kirwan soiling classification system [25] and the Wexner Continence Grading Scale [26], continuing for up to three years following the surgery.

### 2.5. Sample Size Calculation

Previous studies have reported a recurrence rate of 13% following anal sphincter-preserving surgery for T1 and T2 stage rectal cancer cases. Specifically, for patients with pT1 rectal cancer, the JSCCR colorectal cancer registry documented a recurrence rate of 7.4% [23,27]. Based on these findings, the threshold for the five-year recurrence-free survival rate was determined to be 87%, while the anticipated rate was set at 95%, reflecting the expected improvement in outcomes with the applied treatment protocols. A one-sided significance test was employed because we hypothesized that the treatment would only result in an improvement in the recurrence-free survival rate rather than a detrimental effect. With a one-sided significance level of 5% and statistical power of 80%, the minimum required sample size was calculated to be 45 patients. To accommodate for potential dropouts or withdrawals, the target enrollment was increased to at least 50 patients, ensuring that this study would maintain sufficient power despite any attrition during the follow-up period.

### 2.6. Statistical Analysis

In this study, the dataset included all cases that were properly registered, with any duplicate or erroneous entries being carefully excluded to ensure the accuracy of the analysis. Survival curves were generated using the Kaplan–Meier method, which provides a clear visualization of survival probabilities over time. To assess the significance of differences between survival curves, the log-rank test was applied, offering a robust comparison across different patient groups. All statistical analyses were performed using JMP software (JMP Pro 17.0.0, SAS Institute, Cary, NC, USA), a widely recognized tool for comprehensive data analysis. Statistical significance was assessed through various tests depending on the type of data: for ordered categorical variables, the chi-squared test was employed; for survival data, the log-rank test was used; and for continuous variables, the Mann–Whitney U test was applied to compare distributions. A *p*-value of less than 0.05 was considered to indicate statistical significance, ensuring that the findings were robust and reliable.

## 3. Results

### 3.1. Patient Background

This study included a total of 52 patients, with 36 patients diagnosed with T1 and 16 patients diagnosed with T2 lower rectal cancer. Patient backgrounds are presented in Table 1. Significant differences were observed between the submucosal (SM) and muscularis propria (MP) invasion groups in terms of age, venous invasion, and surgical approach.

### 3.2. Primary and Secondary Endpoints

**Figure 1:**
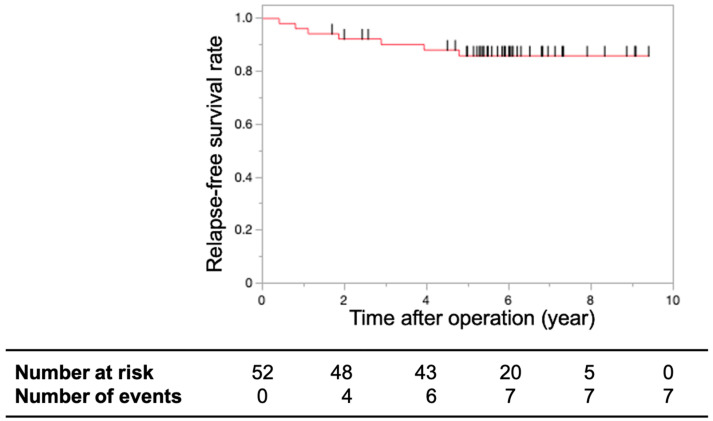
Relapse-free survival rates. Kaplan–Meier curve showing the relapse-free survival rates of patients with T1 or T2 lower rectal cancer treated with CRT using S-1 after limited surgery. The three-year and five-year relapse-free survival rates were 90.17% and 85.87%, respectively. The short black lines on the survival curve indicate censored data.

The three-year and five-year RFS rates, which were the primary endpoints, were 90.17% and 85.87%, respectively (Figure 1).

**Figure 2:**
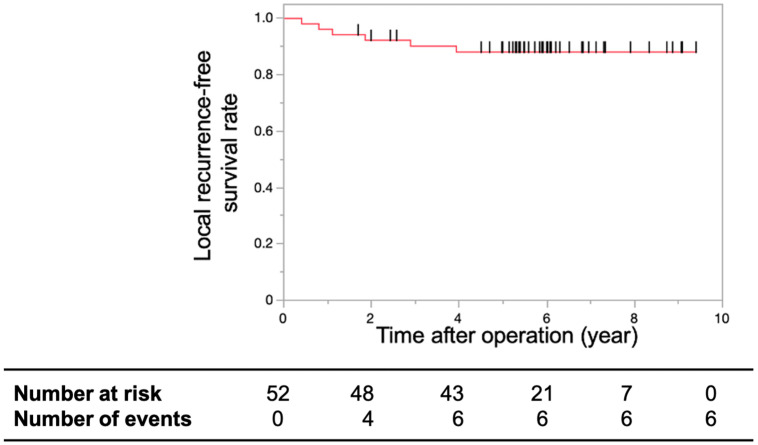
Local recurrence-free survival rates. Kaplan–Meier curve showing the three-year and five-year local recurrence-free survival rates of patients with T1 or T2 lower rectal cancer treated with CRT using S-1 after limited surgery. The three-year and five-year local recurrence-free survival rates were 90.17% and 88.07%, respectively. The short black lines on the survival curve indicate censored data.

Median follow-up time was 70.18 months (interquartile range, 60.51–83.48). The three- and five-year LRFS rates were 90.17% and 88.07%, respectively (Figure 2).

**Figure 3:**
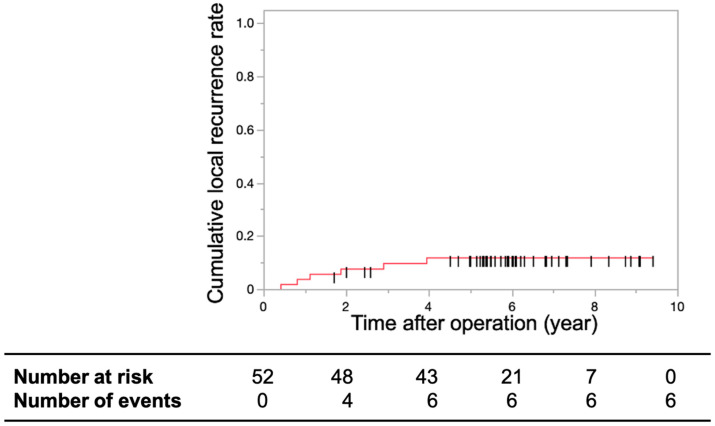
Cumulative local recurrence rates. Kaplan–Meier curve representing the cumulative local recurrence rates in patients with T1 or T2 lower rectal cancer treated with CRT using S-1 after limited surgery over a period of five years. The three-year and five-year local relapse rates were 9.83% and 11.93%, respectively. The short black lines on the survival curve indicate censored data.

**Figure 4:**
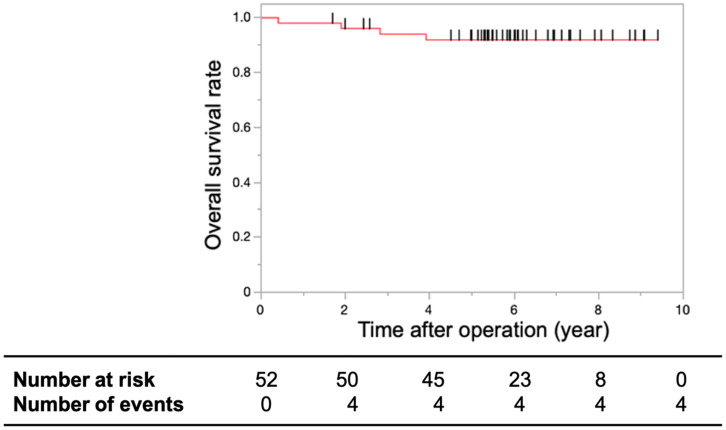
Overall survival rates. Kaplan–Meier curve depicting the overall survival rates of patients with T1 or T2 lower rectal cancer treated with CRT using S-1 after limited surgery. The overall survival rates at three and five years were 94.03% and 91.94%, respectively. The short black lines on the survival curve indicate censored data.

The cumulative local recurrence rates are shown in Figure 3. The 3-year local recurrence rate was 9.83%, and the 5-year local recurrence rate was 11.93%. The anal sphincter preservation rates are shown in Figure 4.

Table 2 details the adverse events of CRT and recurrence rates for both the M.S. and MP groups. Out of 16 cases of MP cancer, recurrence was observed in 4 cases (26.7%).

In contrast, among 36 cases of SM cancer, recurrence was observed in two cases (6.1%) (Table 3).

The OS rates at three and five years were 94.03% and 91.94%, respectively (Figure 4).

**Figure 5:**
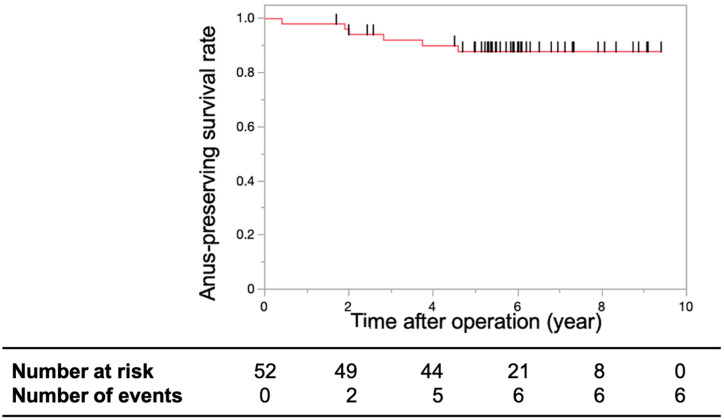
Anal sphincter preservation rate. Kaplan–Meier curve representing the anal-preserving survival rates in patients with T1 or T2 lower rectal cancer treated with CRT using S-1 after limited surgery over a period of five years. The three-year and five-year local recurrence rates were 92.06% and 87.83%, respectively. The short black lines on the survival curve indicate censored data.

The 3-year anal sphincter preservation rate was 92.06%, and the 5-year anal sphincter preservation rate was 87.83% (Figure 5).

**Figure 6:**
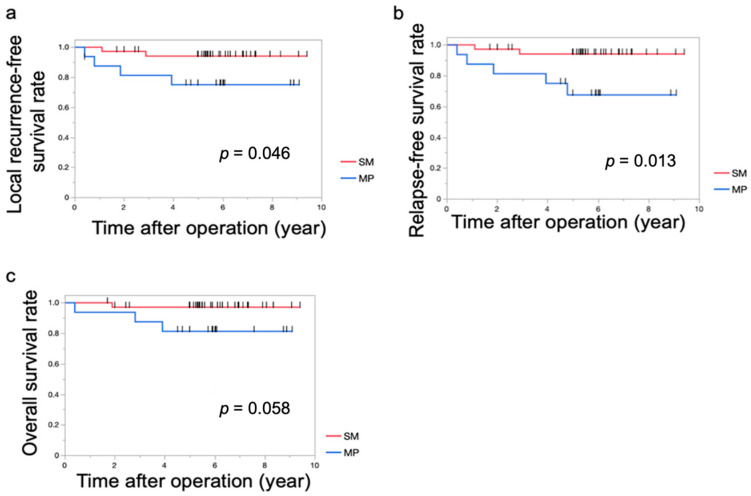
Survival and recurrence rates stratified by SM and MP invasion. (**a**) Kaplan–Meier curves for local recurrence-free survival stratified by SM and MP invasion. (**b**) Kaplan–Meier curves for relapse-free survival stratified by SM and MP invasion. (**c**) Kaplan–Meier curves for overall survival stratified by SM and MP invasion. The MP group exhibited significantly worse local recurrence-free survival and relapse-free survival than the SM group. Differences in survival were assessed using the log-rank test, and *p* values less than 0.05 were considered statistically significant (less than 0.05 are underlined). The short black lines on the survival curve indicate censored data.

Cox regression analysis was performed to investigate the association between clinicopathological factors and RFS (Table 4). Figure 6 presents the LRFS, RFS, and OS, respectively, stratified by the M.S. and MP groups.

Notably, the MP group demonstrated significantly worse LRFS, and RFS compared to the SM group. Among the 16 cases of MP cancer, distant metastasis was observed in two cases (Table 5). In contrast, no distant metastasis was observed in the cases of SM cancer.

## 4. Discussion

Transanal local excision, also referred to as transanal minimally invasive surgery, is an alternative for early cancer treatment located at the lower rectum, with a high expectation for preserving anal function, offering significantly lower rates of morbidity and mortality compared to radical surgeries like low anterior resection or abdominoperineal resection [28]. Addressing concerns related to local recurrence, transanal minimally invasive surgery can be conducted by well-experienced surgeons, leading to high-quality local excision of early rectal cancer with low rates of positive margins [29]. Postoperative complications are relatively infrequent, with bleeding occurring in 1.7–2.7% of cases and pelvic abscesses in 1–2.7% [30]. In our study, no severe postoperative complications were reported, consistent with previous findings [29,31].

The completion rate of treatment in the present study was 76.9% [22], which is lower compared to rates reported in previous prospective studies [20,32,33,34]. In a similar trial, patients with T1 or T2 lower rectal cancer who underwent local resection followed by 45 Gy of radiation therapy and continuous intravenous infusion of 5-fluorouracil or oral administration of capecitabine showed completion rates ranging from 82 to 86% [20,32]. For neoadjuvant CRT, previous studies using S-1 for locally advanced rectal cancer showed completion rates between 86.5 and 91% [33,34]. In our study, compliance rates were 88.4% for chemotherapy and 82.7% for radiotherapy. These rates might have influenced the primary endpoint due to patient-requested treatment discontinuation, even for Grade 2 or lower adverse events. A Grade 3 adverse event (stomatitis) was observed in only 1 out of 52 cases in this study, which is better than the rates in previous CRT studies using S-1 [33,34]. This is attributed to early treatment cessation upon the occurrence of Grade 2 adverse events or at the patient’s request, which may have ultimately had a negative impact on the primary endpoint. This study presented long-term results of combining local excision with CRT using S-1 in T1 or T2 lower rectal cancer patients. The three-year and five-year RFS rates were 90.17% and 85.87%, respectively. OS rates at three and five years were 94.03% and 91.94%, respectively. LRFS rates at three and five years were 90.17% and 88.07%, respectively. As previously reported, there was no deterioration in anal function in our study’s surgical procedures [20,35,36,37,38]. This combined treatment method provides favorable long-term outcomes, particularly for T1 lesions. Earlier studies indicated that local excision alone was considered sufficient for completely resected, differentiated (including well and moderately differentiated) pT1 cases with no lymphovascular invasion [39,40]. However, in our study, we aimed to evaluate the long-term outcomes of chemoradiotherapy (CRT) with S-1 following limited surgery. This approach is underscored by more recent evidence suggesting the potential benefits of adjuvant therapy in reducing recurrence rates, especially in higher-risk patients, such as those with deep submucosal invasion or high tumor budding. Additionally, our research has identified cases with positive vascular invasion, which have shown recurrence. We have thus incorporated the examination of risk factors for recurrence, highlighting a significant distinction from past reports.

## 5. Limitations

The sample size was relatively small, and deviations from the protocol treatment schedule could have affected treatment completion rates and CRT-related adverse events. Additionally, genetic information such as MSI, RAS, and BRAF mutation status was not collected for this study. We acknowledge that these molecular factors are crucial in current treatment approaches, and this is a limitation of our study. Future studies with larger cohorts and strict adherence to protocol schedules are needed to validate our findings. Regarding M.S. cancer, the results of the single-arm confirmatory trial JCOG1612 [41], which investigated the efficacy of radiotherapy combined with capecitabine for high-risk submucosal invasive lower rectal cancer (pT1) with negative vertical margins following local excision, are highly anticipated. This study aims to provide valuable insights into the management of high-risk submucosal invasive cancers, particularly by evaluating the effectiveness of current treatment modalities such as CRT with S-1 after limited surgery for T1 lower rectal cancer. By analyzing outcomes such as recurrence, survival, and local control rates, we hope to identify therapeutic strategies that not only improve patient outcomes but also preserve critical functions like anal continence. Furthermore, the findings from this study may contribute to the development of more tailored approaches for patients with higher-risk profiles, such as those with stage T2 cancers, where additional treatment may be necessary to optimize results. Additionally, a multicenter randomized trial (TESAR trial) comparing radical surgery with adjuvant CRT after local excision for high-risk T1 and low-risk T2 rectal cancers is currently ongoing [42].

## 6. Conclusions

Adjuvant CRT using S-1 after limited surgery demonstrated acceptable long-term outcomes and safety profiles in patients with T1 lower rectal cancer, with better relapse-free and OS rates, as well as anal function. However, the higher recurrence rates observed for patients with T2 lower rectal cancer suggest that additional or alternative treatment strategies should be considered.

## Figures and Tables

**Table 1:** Patient characteristics.

	SM, N = 36 (%)	MP, N = 16 (%)	*p* Value
Age, median (IQR) [<65/≥65]	64 (54–68) 21 (58.3%)/15 (41.7%)	70.5 (65–75) 4 (25.0%)/12 (75.0%)	0.004 * 0.037 **
Sex [Male / Female]	20 (55.6%)/16 (44.4%)	7 (43.7%)/8 (56.3%)	0.551
ECOG-PS [0/1]	33 (100%)/0 (0%)	14 (87.5%)/2 (12.5%)	0.102
Location [Rb/P]	35 (97.1%)/1 (2.9%)	15 (93.7%)/1 (6.3%)	1.000
Histological type [tub1/tub2]	18 (51.4%)/17 (48.6%)	6 (40.0%)/9 (60.0%)	0.545
Greatest diameter (median/IQR) [<22/≥22]	20 (18–25) 17 (50.0%)/17 (50.0%)	23.5 (20–27) 6 (37.5%)/10 (62.5%)	0.321 * 0.546 **
Lymphatic invasion [+/−]	11 (30.6%)/25 (69.4%)	8 (50.0%)/8 (50.0%)	0.220
Venous invasion [+/−]	13 (36.1%)/23 (63.9%)	11 (68.8%)/5 (31.2%)	0.038
Budding grade [1/2–3]	23 (79.3%)/6 (20.7%)	1 (25.0%)/3 (75.0%)	0.052
Preoperative CEA, median (IQR) (ng/mL) [<2.0/≥2.0]	1.95 (1.4–2.78) 18 (50.0%)/18 (50.0%)	2.1 (1.55–3.23) 6 (37.5%)/10 (62.5%)	0.579 0.549 **
Preoperative CA19-9, median (IQR) (ng/mL) [<6.5/≥6.5]	6.5 (3.78–12.75) 18 (50.0%)/18 (50.0%)	6.5 (3.4–9.75) 9 (56.3%)/7 (43.7%)	0.960 * 1.000 **
Operation approach (Transanal excision/EMR or ESD)	21 (58.3%)/15 (41.7%)	16 (100%)/0 (0%)	0.002
Operation time median (IQR) [<68/≥68]	82 (45.3–99.5) 13 (43.3%)/17 (56.7%)	59.5 (35.3–74.5) 10 (62.5%)/6 (37.5%)	0.134 * 0.353 **

* Mann–Whitney U test was performed. ** The chi-square-test was performed. SM, submucosal invasion; MP, muscularis propria invasion; ECOG-PS, Eastern Cooperative Oncology Group Performance Status scale; Rb, lower rectum; P, anal canal; tub1, well-differentiated adenocarcinoma; tub2, moderately differentiated adenocarcinoma; CEA, carcinoembryonic antigen; CA19-9, carbohydrate antigen 19-9; EMR, endoscopic mucosal resection; ESD, endoscopic submucosal dissection.

**Table 2:** Adverse events and recurrence in 52 patients.

	SM, N = 36 (%)	MP, N = 16 (%)	*p* Value
Adverse events (Grade 3–4) [+/−]	0 (0%)/36 (100%)	1 (6.2%)/15 (93.8%)	0.3077
Locoregional recurrence [+/−]	2 (6.1%)/31 (93.9%)	4 (26.7%)/11 (73.3%)	0.0672
Regional lymph node recurrence	0 (0%)	1 (6.2%)	NA

SM, submucosal invasion; MP, muscularis propria invasion; NA, not available.

**Table 3:** Patient background in cases with locoregional recurrences.

Case No.	Age	Sex	PS	Medical History	CEA (ng/mL)	CA19-9 (U/mL)	Surgery	Op time (min)	Blood Loss (mL)	Location	Tumor Size (mm)	Histology	Tumor Invasion	Ly	V	BD	Genetic Information
1	62	F	0	None	2.2	7	TAE	69	0	Rb	27 × 15	tub2	MP	0	2	NA	RAS-mt (KRAS G13D)/BRAF-wt/MSS
2	70	F	0	Varicose veins	0.9	<2	TAE	35	40	Rb	25 × 20	tub2	MP	0	2	NA	NA
3	60	F	0	Hypertension, appendicitis	1.5	8	TAE	52	0	Rb	23 × 22	tub2	MP	0	3	2	RAS-mt
4	49	M	0	Anal fistula	3.9	15	TAE	97	0	Rb	20 × 20	tub2	SM	1	3	1	RAS-wt/BRAF-wt/MSS
5	73	M	0	Asthma, AF	2.4	<2	TAE	67	0	Rb	13 × 13	tub1	MP	0	1	NA	MSS
6	54	F	0	None	1.1	14	ESD	107	5	Rb	55 × 29	tub2	SM.	1	0	1	RAS-mt

PS, Eastern Cooperative Oncology Group Performance Status scale performance status; CEA, carcinoembryonic antigen; CA19-9, carbohydrate antigen 19-9; Op time, operative time; Ly, pathologically defined lymphatic invasion according to the Japanese classification of colorectal, appendiceal, and anal carcinoma; V, pathologically defined venous invasion according to the Japanese classification of colorectal, appendiceal, and anal carcinoma; BD, budding grade according to the Japanese classification of colorectal, appendiceal, and anal carcinoma; F, female; M, male; AF, arterial fibrillation; TAE, transanal excision; ESD endoscopic submucosal dissection; Rb, lower rectum; tub1, well-differentiated adenocarcinoma; tub2, moderately differentiated adenocarcinoma; SM, submucosal invasion; MP, muscularis propria invasion; NA, not available; mt, mutant type; wt, wild type, MSS, microsatellite stable.

**Table 4:** Cox regression analysis of clinicopathological factors and relapse-free survival.

Factors	HR	95% CI	*p* Value
Age [≥65/<65]	0.697	0.156–3.116	0.637
Sex [Male/Female]	0.706	0.317–6.330	0.649
Tumor invasion [MP/M.S.]	6.178	1.197–31.890	0.030
Lymphatic invasion [+/−]	4.722	0.568–39.240	0.151
Venous invasion [+/−]	2.863	0.555–14.761	0.209
CEA [≥2.0/<2.0]	1.152	0.258–5.149	0.853
CA19-9 [≥6.5/<6.5]	1.39	0.311–6.217	0.666

HR, hazard ratio; 95% CI, 95% confidence interval; M.S., submucosal invasion; MP, muscularis propria invasion; CEA, carcinoembryonic antigen; CA19-9, carbohydrate antigen 19-9.

**Table 5:** Patient background in recurrence cases.

Case No.	TS-1 Dosage (mg)	Dose Reduction or Discontinuation of Treatment	Complication (CD III or IV)	Recurrence Site	RFS (Month)	Survival Outcome	OS (Month)
1	80	Discontinuation of TS-1 due to leukopenia (G2) *	None	Bilateral iliac lymph node	57	Survival	69
2	120	None	None	Local	47	Survival	57
3	120	None	None	Local, lung, pelvic lymph node	9	Death	33
4	120	None	None	Local	34	Death	46
5	120	None	None	Local	22	Death	45
6	100	None	None	Local	13	Death	22

* Adverse events were assessed according to the Common Toxicity Criteria of the National Cancer Institute (version 4.0). RFS, relapse-free survival time; OS, overall survival time.

## Data Availability

The data supporting the findings of this study can be obtained from the corresponding authors, upon reasonable request.
